# Statin-Sensitive Akt1/Src/Caveolin-1 Signaling Enhances Oxidative Stress Resistance in Rhabdomyosarcoma

**DOI:** 10.3390/cancers16050853

**Published:** 2024-02-20

**Authors:** Silvia Codenotti, Leonardo Sandrini, Delia Mandracchia, Luisa Lorenzi, Giovanni Corsetti, Maura Poli, Michela Asperti, Valentina Salvi, Daniela Bosisio, Eugenio Monti, Stefania Mitola, Luca Triggiani, Michele Guescini, Enrico Pozzo, Maurilio Sampaolesi, Stefano Gastaldello, Matteo Cassandri, Francesco Marampon, Alessandro Fanzani

**Affiliations:** 1 Department of Molecular and Translational Medicine, University of Brescia, 25123 Brescia, Italy; leonardo.sandrini@unibs.it (L.S.); delia.mandracchia@unibs.it (D.M.); maura.poli@unibs.it (M.P.); michela.asperti@unibs.it (M.A.); valentina.salvi@unibs.it (V.S.); daniela.bosisio@unibs.it (D.B.); eugenio.monti@unibs.it (E.M.); stefania.mitola@unibs.it (S.M.); 2 Department of Molecular and Translational Medicine, University of Brescia, and ASST Spedali Civili di Brescia, 25123 Brescia, Italy; luisa.lorenzi@unibs.it; 3 Department of Clinical and Experimental Sciences, University of Brescia, 25123 Brescia, Italy; giovanni.corsetti@unibs.it; 4 Department of Radiation Oncology, ASST Spedali Civili di Brescia, University of Brescia, 25123 Brescia, Italy; luca.triggiani@unibs.it; 5 Department of Biomolecular Sciences, University of Urbino Carlo Bo, 61029 Urbino, Italy; michele.guescini@uniurb.it; 6 Department of Development and Regeneration, KU Leuven, 3000 Leuven, Belgium; enrico.pozzo@kuleuven.be (E.P.); maurilio.sampaolesi@kuleuven.be (M.S.); 7 Department of Physiology and Pharmacology, Karolinska Institutet, 17177 Stockholm, Sweden; stefano.gastaldello@ki.se; 8 Department of Radiological Sciences, Oncology and Anatomic Pathology, “Sapienza” University of Rome, 00161 Rome, Italy; matteo.cassandri@uniroma1.it (M.C.); francesco.marampon@uniroma1.it (F.M.)

**Keywords:** Akt1, catalase, caveolin-1, radioresistance, rhabdomyosarcoma, Src

## Abstract

**Simple Summary:**

Treatment of relapsed or metastatic rhabdomyosarcoma (RMS) has low survival rates due to resistance mechanisms. In this work, experiments were undertaken to identify potential druggable pathways involved in radiotherapy resistance. We found that prolonged activation of a protein network formed by Akt1, Src, and caveolin-1 (Cav1) lowers intracellular reactive oxygen species (ROS) levels through the acquisition of high catalase expression, conferring radioresistance to RMS cells. Treatment of radioresistant cells with statins, drugs used worldwide for the treatment of hypercholesterolemia, significantly attenuated the Akt1/Cav1 signaling and radioresistance mechanisms through increased cell apoptosis. This evidence suggests that administration of statins could help to improve the success of radiotherapy in RMS.

**Abstract:**

Identifying the molecular mechanisms underlying radioresistance is a priority for the treatment of RMS, a myogenic tumor accounting for approximately 50% of all pediatric soft tissue sarcomas. We found that irradiation (IR) transiently increased phosphorylation of Akt1, Src, and Cav1 in human RD and RH30 lines. Synthetic inhibition of Akt1 and Src phosphorylation increased ROS levels in all RMS lines, promoting cellular radiosensitization. Accordingly, the elevated activation of the Akt1/Src/Cav1 pathway, as detected in two RD lines characterized by overexpression of a myristoylated Akt1 form (myrAkt1) or Cav1 (RDCav1), was correlated with reduced levels of ROS, higher expression of catalase, and increased radioresistance. We found that treatment with cholesterol-lowering drugs such as lovastatin and simvastatin promoted cell apoptosis in all RMS lines by reducing Akt1 and Cav1 levels and increasing intracellular ROS levels. Combining statins with IR significantly increased DNA damage and cell apoptosis as assessed by γ histone 2AX (γH2AX) staining and FACS analysis. Furthermore, in combination with the chemotherapeutic agent actinomycin D, statins were effective in reducing cell survival through increased apoptosis. Taken together, our findings suggest that the molecularly linked signature formed by Akt1, Src, Cav1, and catalase may represent a prognostic determinant for identifying subgroups of RMS patients with higher probability of recurrence after radiotherapy. Furthermore, statin-induced oxidative stress could represent a treatment option to improve the success of radiotherapy.

## 1. Introduction

RMS is a pediatric tumor divided into fusion-positive (FPRMS) and fusion-negative (FNRMS) subtypes depending on the presence or absence of t(2;13) or t(1;13) chromosomal translocations [1]. Multiagent chemotherapy (vincristine, actinomycin D, and cyclophosphamide or ifosfamide) followed by radiotherapy represents the standard treatment of RMS patients. Although this approach results in >70% overall 5-year survival for localized tumors, intrinsic or acquired radioresistance is a predictor of poor (less than 30%) survival [2,3]. Deregulation of receptor tyrosine kinase (RTK) signaling through the RAS/extracellular signal-regulated kinases (ERK) and serine/threonine Akt/mammalian target of rapamycin (mTOR) pathways plays a key role in the pathogenesis of RMS [4]. Enhanced signaling through these pathways influences various cellular functions, including proliferation, migration, and invasion [5]. In particular, the Akt pathway, which induces both genome instability and DNA damage repair [6], displays oncogenic and antiapoptotic activities [7]. In RMS, Akt activation is negatively associated with patient survival [8]. Recently, we demonstrated that stable expression of an active myrAkt1 form in human RD cells correlates with enhanced DNA damage repair via activation of DNA-dependent protein kinase (DNA-PK) [9]. In a significant proportion of RMS cases, members of the non-receptor tyrosine kinase Src family are found to be abnormally activated [10,11], often promoting resistance mechanisms [12,13,14]. Interestingly, the scaffolding protein Cav1, a cholesterol-binding protein involved in the formation of caveolar lipid rafts [15,16] that also plays a role in RMS malignancy [17,18,19], is a well-known target of the Src family [20]. Src-mediated phosphorylation of Cav1 on tyrosine-14 activates a variety of pathways related to cancer aggressiveness, influencing cell migration, invasion, and resistance to oxidative stress [21,22]. In recent years there has been growing interest in the mechanisms underlying radiation resistance in RMS [3]. In previous works, we demonstrated that increases in Cav1 and Akt levels support radioresistance [9,23]. Furthermore, their expression was increased in radioresistant RD and RH30 lines developed with a hypo-fractionated radiotherapy protocol [24]. The experiments in this work were undertaken to evaluate whether and how crosstalk between Akt1, Src, and Cav1 may influence oxidative stress levels and response to IR treatment. We additionally investigated whether inhibition of the mevalonate pathway by statins (which causes reduction of isoprenoid intermediates and cholesterol [25]) could affect the Akt1/Src/Cav1 signaling network and oxidative stress, thereby influencing radioresistance mechanisms. In this context, we evaluated whether the combination of statins with IR or actinomycin D could increase the efficacy of therapy. For this purpose, we used human RD and RH30 lines, representative of FNRMS and FPRMS, respectively, as well as previously generated RD lines characterized by radioresistant traits due to their respective overexpression of myrAkt1 [9] and Cav1 [23].

## 2. Materials and Methods

### 2.1. Bioinformatic Analysis

RNA sequencing (RNA-seq) data of RMS patient cohort and normal skeletal muscle samples were downloaded from the NCBI Gene Expression Omnibus database (accession number GSE108022) [4]. RNA-seq data were preprocessed and normalized using the iDEP.96 tool (Prof. Xijin Ge, South Dakota State University, Brookings, SD, USA). [26], and gene expression was calculated as Log2 Counts per Million (CPM). Data were analyzed and plotted using GraphPad Prism 8.0 software (GraphPad Software, San Diego, CA, USA).

### 2.2. Reagents and Antibodies

The compounds PD098059, LY294002, PP2, lovastatin, simvastatin, and actinomycin D were from Sigma Aldrich (Milan, Italy). MK2206 was from Aurogene (Rome, Italy). All compounds were dissolved in dimethyl sulfoxide (DMSO) vehicle. Unless otherwise stated, PD098059, LY294002, MK2206, and lovastatin were used at 10 µM concentration. PP2 was used at 20 µM, simvastatin at 5 µM, and actinomycin D at 0.5 nM concentration. The following primary antibodies were used: total and phosphorylated Akt1 (Ser473) (MAB-9432 and MAB-94111, Immunological Sciences, Rome, Italy), total and phosphorylated Src (Tyr416) (AB-84358, Immunological Sciences, Rome, Italy; #2101, Cell Signaling Technology, Danvers, MA, USA), total and phosphorylated Cav1 (Tyr14) (AB-81830, Immunological Sciences, Rome, Italy; 611338, BD, Milan, Italy), total and phosphorylated Erk1/2 (Thr202/Tyr204) (sc-135900 and sc-7383, Santa Cruz Biotechnology, Dallas, TX, USA), catalase (sc-271803, Santa Cruz Biotechnology, Dallas, TX, USA), phosphorylated histone-2AX (H2AX) (Ser139) (#9718, Cell Signaling Technology, Danvers, MA, USA), and α-tubulin (T5168, Sigma Aldrich, Milan, Italy). Secondary anti-mouse (IS20400) and anti-rabbit (IS20402) antibodies were from Immunological Sciences (Rome, Italy).

### 2.3. Cell Cultures

All cell lines were maintained in a humified incubator at 37 °C with 5% CO_2_ and cultured in high-glucose Dulbecco’s Modified Eagle’s Medium (DMEM) (Euroclone, Milan, Italy) supplemented with 100 mg/mL penicillin/streptomycin and 10% fetal bovine serum (FBS) (Euroclone, Milan, Italy). Human RD and RH30 cells were purchased from the European Collection of Cell Cultures (ECACC). Stable RDCav1 and myrAkt1 clones were established in our previous studies [9,23]. All the cell lines were routinely tested for mycoplasma contamination.

### 2.4. ROS Measurement

ROS production was quantified using an CM-H2DCFDA probe (ThermoFisher Scientific, Milan, Italy) according to the manufacturer’s instructions. Cells were plated in black 96-well plates and treated with the indicated compounds. After 24 h, the supernatant was removed and cells were incubated with medium containing 1 μM ROS probe for 30 min at 37 °C protected from light. The medium containing the probe was then removed and replaced with fresh medium. The fluorescence was monitored using the EnSight Multimode plate reader (PerkinElmer, Waltham, MA, USA) at 492 nm excitation/517 nm emission wavelengths. Cells were then fixed in paraformaldehyde (PFA) solution (3% PFA in PBS) for 20 min at 4 °C and processed for the crystal violet assay used as normalization. Briefly, cells were stained with crystal violet solution (0.2% crystal violet in PBS with 20% methanol) for 10 min at room temperature (RT) and washed with deionized water, then the dye was solubilized in sodium dodecyl sulfate (SDS) solution (1% SDS in PBS) by shaking the plates until complete dissolution was achieved. Absorbance was measured by reading the plate at 595 nm emission wavelength. Results were expressed as the ratio of ROS fluorescence/crystal violet absorbance and reported as the fold change, setting control to 1.

### 2.5. IR Treatments

IR treatment was performed at RT on subconfluent cells pre-treated or not with the indicated compounds for 2 h at 4 Gray (Gy) doses using an X-ray linear accelerator (dose rate of 2 Gy/min). Post-irradiated cells were processed for immunoblotting, immunofluorescence, or clonogenic assays.

### 2.6. Immunoblotting Analysis

Cells were lysed in cold RIPA buffer (20 mM Tris-HCl at pH 7.6, 1% Nonidet P40, 0.5% sodium deoxycholate, 0.1% SDS, 50 mM NaCl) supplemented with phosphatase inhibitors (1 mM Na_3_VO_4_ and 4 mM NaF) and a cocktail of protease inhibitors (Roche, Milan, Italy). Whole-cell lysates were sonicated prior to centrifugation (12,000× *g*, 10 min at 4 °C), then the protein concentration was determined by Bradford assay. Equal protein amounts were boiled at 99 °C for 5 min before SDS-PAGE, followed by transfer to polyvinylidene fluoride (PVDF) membranes. Then, membranes were blocked with 5% milk in TBS with 0.1% Tween-20 (TBS-T) for 15 min at RT prior to incubation with primary antibody overnight at 4 °C. Following TBS-T washes, membranes were incubated with HRP-conjugated secondary antibody for 1 h at RT and then TBS-T washed again. Proteins were detected using enhanced chemiluminescence (ECL) reagent (Euroclone, Milan, Italy). Band densitometry (Supplementary File S1) was calculated using Gel Pro Analyzer 4 software (MediaCybernetics Inc., Rockville, MD, USA) and expressed by arbitrary density units, setting control to 1.

### 2.7. Clonogenic Assay

Post-irradiated cells were detached with trypsin and reseeded in triplicates into 6-well plates (at a density of 500 for myrAkt1 and RDCav1 or 1000 for RD and RH30) to evaluate clonogenic survival. Plates were incubated for 10 days under standard conditions, then grown colonies were fixed with PFA solution (20 min at 4 °C) and stained with crystal violet solution (0.2% crystal violet in PBS with 20% methanol) for 10 min at RT. Pictures were taken after washing with deionized water and the number of colonies was counted. The survival fraction was calculated by dividing the number of colonies by the number of seeded cells and normalized on the plating efficiency of the non-irradiated controls.

### 2.8. Immunofluorescence Analysis

Cells seeded onto 12 mm glass coverslips were fixed with PFA solution (3% PFA in PBS) for 20 min at 4 °C, permeabilized with Triton X-100 (0.1% Triton X-100 in PBS) (10 min at RT), and blocked with bovine serum albumin (BSA) solution (1% BSA in PBS with 0.1% sodium azide) for 30 min at RT prior to incubation with primary antibody for 3 h at RT. Following BSA washes, cells were incubated with secondary antibody for 45 min at RT while protected from light. Nuclei were counterstained with Hoechst dye (30 s at RT) and samples were mounted on slides using Mowiol mounting media. Images were acquired by a fluorescence Axiovert microscope (Carl Zeiss, Oberkochen, Germany) at 63× magnification using ImagePro Plus software (Media Cybernetics, Inc. Rockville, MD, USA). Quantification of nuclear γH2AX-positive cells was obtained by counting the number of cells showing nuclear γH2AX foci normalized on the total cell number in ten different fields.

### 2.9. PCR Analysis

RNA from proliferating cells was reverse transcribed using a high-capacity cDNA reverse transcription kit (Applied Biosystem, Foster City, CA, USA). Standard semi-quantitative PCR was performed using the primers 5’-CGTCTGGGGGCAAATACGTAGACT-3’ and 5’-CTTTCTGCAAGTTGATGCGGACATTGCTGAAT-3’ for the CAV1 fragment (537 bp) and 5’-CGTGGAGTCTACTGGCGTCCTTC-3’ and 5’-AACCACGAGAAATATGACACACTCCC -3’ for the GAPDH fragment (267 bp). Band densitometry was calculated using Gel Pro Analyzer 4 software (MediaCybernetics Inc., Rockville, MD, USA) and expressed by arbitrary density units, setting control to 1.

### 2.10. Flow Cytometric Analysis

Cell apoptosis was assessed using an Annexin V/Propidium Iodide (PI) apoptosis detection kit (Immunostep Biotec, Salamanca, Spain) according to the manufacturer’s instructions. Cells were seeded into 6-well plates and treated with the indicated compounds or IR for 72 h. Then, cells were collected in flow cytometry tubes, PBS washed, resuspended in binding buffer, and double stained with Annexin-V-FITC/PI. Cytofluorimetric analysis was performed using a MACSQuant Analyzer (Miltenyi Biotec, Bologna, Italy). Cell debris, doublets, and aggregates were excluded from the analysis, and 10,000 events per sample were analyzed.

### 2.11. Neutral Red Assay

Cells were seeded in 96-well plates prior to administration of the indicated compounds. After 48 h of treatment, the medium was replaced with DMEM supplemented with 5% FBS and 40 μg/mL neutral red dye, then the plates were incubated at 37 °C for 2 h. The cells were then PBS-washed and incubated with a destaining solution (50% ethanol in deionized water with 1% acetic glacial acid). The plates were shaken until complete dye extraction was achieved, then absorbance was measured by reading the plate at 540 nm emission wavelength.

### 2.12. Statistical Analysis

All error bars represent standard deviation. For pairwise comparisons, an unpaired Student’s *t* test was used, whereas a one-way ANOVA test with Tukey’s post hoc test was used to compare the means among three or more groups using GraphPad Prism 8 software (GraphPad Software, San Diego, CA, USA). The significance threshold was a *p*-value < 0.05.

## 3. Results

### 3.1. Activation of the Akt1/Src/Cav1 Axis Promotes Cell Survival in Post-Irradiated RD and RH30 Lines

**Figure 1:**
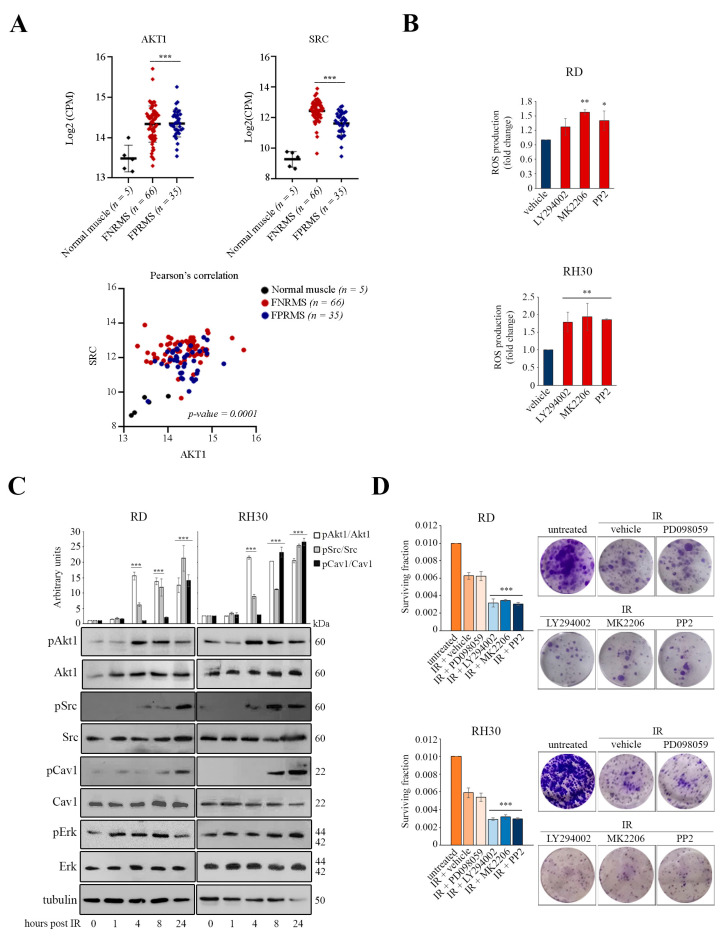
Involvement of the Akt1, Src, and Cav1 proteins in oxidative stress and radiation response in RMS lines. (**A**) Quantification of AKT1 and SRC gene expression was performed using an RNA-seq dataset (GSE108022) relative to human FNRMS (*n* = 66), FPRMS (*n* = 35), and normal skeletal muscle (*n* = 5) samples (top panels). Each point represents an individual sample. The mean expression is highlighted by a black horizontal bar. *** *p*-value < 0.0001; one-way ANOVA test vs. normal skeletal muscle. Correlation between AKT1 and SRC expression was calculated by Pearson’s coefficient (bottom panel). (**B**) RD and RH30 cells (3 × 10^3^) were plated in black 96-well plates in triplicates 24 h prior to treatment with the indicated compounds. Quantification of ROS production with the CM-H2DCFDA probe was performed after 24 h (*n* = 2). Data are mean ± SEM, * *p*-value < 0.05; ** *p*-value < 0.001; one-way ANOVA test vs. vehicle-treated cells. (**C**) RD and RH30 cells (1.5 × 10^5^) were seeded into 60mm dishes. After 24 h, cells were irradiated and collected at the indicated time points for IB (*n* = 2). Densitometric quantifications are reported in the top graph after tubulin normalization. Data are mean ± SEM, *** *p*-value < 0.0001; one-way ANOVA test vs. 0 h time point. (**D**) Subconfluent RD and RH30 cells were pretreated for 2 h with the indicated compounds prior to IR. Cell survival was evaluated by clonogenic assay (*n* = 3). Data are mean ± SEM, *** *p*-value < 0.0001; one-way ANOVA test vs. vehicle-treated irradiated cells. Representative images were taken after crystal violet incorporation.

The Akt and Src pathways are abnormally activated in RMS [8,10]. By performing in silico analysis of RNA-seq data (GEO: GSE108022), we found that both AKT1 and SRC are significantly more expressed in human RMS samples, regardless of subtype, than in skeletal muscle samples (Figure 1A, top panels) [4]. Moreover, their expression is significantly correlated, as calculated by Pearson’s coefficient (Figure 1A, bottom panel). In light of the important role of these kinases in regulating anti-apoptotic and oxidative stress resistance mechanisms, we wondered whether their inhibition could affect intracellular ROS levels. For this purpose, proliferating human RD and RH30 cells were treated with LY294002, MK2206, and PP2, which are inhibitors of the phosphatidylinositol 3-kinase (PI3K), Akt, and Src kinases, respectively. All these treatments were effective in reducing cell proliferation without affecting cell viability and produced a significant increase in intracellular ROS levels measured after 24 h by ROS probe (CM-H2DCFDA probe) (Figure 1B). Considering these data, we hypothesized that activation of the Akt1/Src pathway may play a role in the detoxification of intracellular ROS. Therefore, we sought to test whether the Akt/Src pathway can sense oxidative stress in response to IR therapy, which is known to cause high ROS accumulation. For this purpose, human RD and RH30 cells were subjected to an IR dose of 4 Gy and then collected at different time points for IB analysis. We found that phosphorylation of Akt1 on Ser473 (pAkt1) and phosphorylation of Src on Tyr416 were significantly increased between 4 and 8 h in the post-irradiated lines (Figure 1C). Between 8 and 24 h after IR, we found increased phosphorylation of Cav1 on Tyr14 (pCav1) (Figure 1C), a scaffolding protein that is a known target of Src kinases. In contrast, Erk1/2 phosphorylation (pErk) was not affected by IR treatment (Figure 1C). These data suggest that IR-induced oxidative stress could activate the Akt/Src pathway to promote cell survival by ROS detoxification. To test this, we evaluated the cell survival of RMS lines treated for 2 h with inhibitors of the PI3K/Akt, Src, and Erk pathways before receiving IR. As assessed by clonogenic assay, the inhibitors LY294002, MK2206, and PP2 were effective in reducing cell survival of RMS lines, whereas in contrast the ERK1/2 phosphorylation inhibitor PD098059 had no radiosensitizing effect (Figure 1D). Taken together, these results suggest that Akt1/Src activation in collaboration with Cav1 may be implicated in a pro-survival program through modulation of oxidative stress.

### 3.2. Akt1/Src/Cav1/Catalase Signaling Promotes Radioresistance by Buffering Oxidative Stress in RD Cells

**Figure 2:**
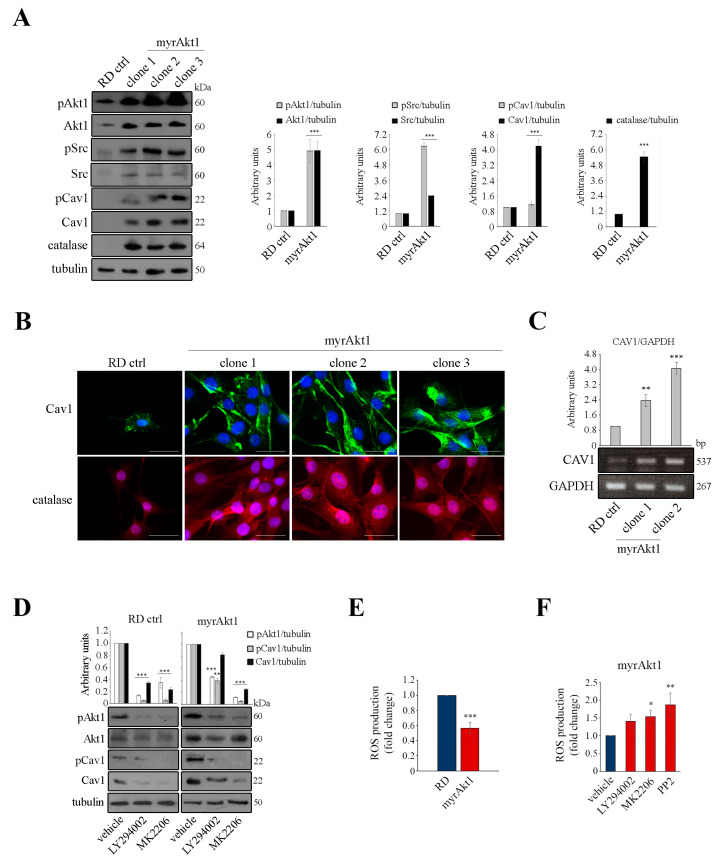
MyrAkt1 signaling drives increased levels of Akt1, Src, Cav1, and catalase, promoting intracellular ROS reduction. (**A**–**C**) Control and myrAkt1 cells (1.5 × 10^5^) were seeded into 60 mm dishes. After 48 h of proliferation, cells were processed for IB (*n* = 2) (**A**), IF (*n* = 3) (**B**), and PCR analyses (*n* = 2) (**C**). Densitometric quantifications are reported in the graphs after tubulin or GAPDH normalization. Data are mean ± SEM, ** *p*-value < 0.001; *** *p*-value < 0.0001; unpaired Student’s *t*-test vs. control RD line. Representative images for IF analysis were taken using a fluorescent microscope at 63 X magnification. The scale bar corresponds to 50 µm. (**D**) After 24 h had elapsed from plating cells (1.0 × 10^5^) into 60 mm dishes, LY294002 and MK2206 or vehicle were provided for up to 72 h before IB analysis (*n* = 2). Densitometric quantifications are reported in the top graph after tubulin normalization. Data are mean ± SEM, ** *p*-value < 0.001; *** *p*-value < 0.0001; one-way ANOVA test vs. vehicle-treated cells. (**E**,**F**) Control and myrAkt1 cells (3 × 10^3^) were plated in black 96-well plates in triplicates 24 h prior to treatment or not with the indicated compounds. Quantification of ROS production with the CM-H2DCFDA probe was performed after 24 h (*n* = 2). Data are mean ± SEM, * *p*-value < 0.05; ** *p*-value < 0.001; *** *p*-value < 0.0001; unpaired Student’s *t*-test vs. control RD line (**E**) or one-way ANOVA test vs. vehicle-treated cells (**F**).

**Figure 3:**
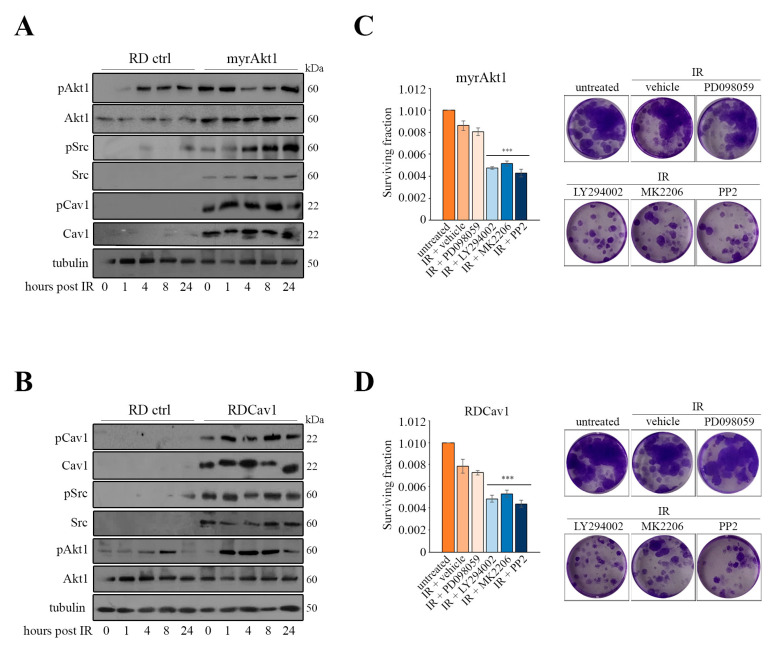
The expression pattern of Akt1, Src, and Cav1 is conserved in the radioresistant myrAkt1 and RDCav1 lines, and its inhibition promotes radiosensitization. (**A**,**B**) IR treatment was performed 24 h after plating control, myrAkt1 and RDCav1 cells (1.5 × 10^5^) in 60 mm dishes. Cells were processed at the indicated time-points for IB (*n* = 2). To explain the absence of Src and Cav1 bands in the control cells, it should be noted that detection was performed under low exposure conditions to avoid interference with the strong signals arisen from protein homogenates of radioresistant cells. Tubulin was used as loading control. (**C**,**D**) Subconfluent myrAkt1 and RDCav1 cells were pretreated for 2 h with the indicated compounds before IR treatment. Cell survival was evaluated by clonogenic assay (*n* = 3). Data are mean ± SEM, *** *p*-value < 0.0001; one-way ANOVA test vs. vehicle-treated irradiated cells. Representative images were taken after crystal violet incorporation.

We wondered whether Akt1 might be involved in the regulation of Src and Cav1. To answer this question, we exploited myrAkt1 cells that exhibit radioresistance hallmarks due to enhanced DNA repair [9]. As demonstrated by IB analysis, in three different RD clones carrying the myrAkt1 form we found significantly increased protein levels of total and phosphorylated Src and Cav1 forms as compared with control RD cells (Figure 2A). The myrAkt1 cells showed elevated expression of catalase (Figure 2A), an enzyme involved in H_2_O_2_ detoxification [27], which we have previously recognized as being highly expressed in RDCav1 cells [23]. After staining the cells with antibodies against Cav1 or catalase, we used IF analysis to confirm the increased expression of these markers in myrAkt1 cells compared with control cells (Figure 2B). We demonstrated by semiquantitative PCR that myrAkt1 increases Cav1 gene transcription (Figure 2C). Following treatment with LY294002 and MK2206, Cav1 protein levels were downregulated in both control and myrAkt1 cells (Figure 2D), suggesting that Cav1 is a downstream target of the PI3K/Akt pathway. In myrAkt1 cells, intracellular levels of ROS measured under basal conditions were lower than those in control RD cells (Figure 2E). Accordingly, treatment of myrAkt1 cells with LY294002, MK2206, and PP2 increased ROS levels as measured after 24 h (Figure 2F), confirming that activation of Akt1 signaling is involved in maintaining low endogenous levels of oxidative stress. Next, we analyzed Akt1/Src/Cav1 signaling in response to IR treatment in myrAkt1 and RDCav1 radioresistant lines. As shown in Figure 3A,B, the post-irradiated cell lines had higher levels of Src and pSrc as well as of Cav1 and pCav1 compared to the controls. Akt1 and pAkt1 levels in myrAkt1 cells increased at baseline, and remained higher than in controls after IR (Figure 3A). In contrast, the total form of Akt1 in RDCav1 cells did not change compared with controls. However, phosphorylation of Akt1 increased more rapidly in RDCav1 cells than in controls after IR (Figure 3B), suggesting that Cav1 may facilitate phosphorylation of Akt1 in response to IR. As demonstrated by clonogenic assay (Figure 3C,D), cell survival of both radioresistant lines was significantly abrogated by pretreatment with LY294002, MK2206, and PP2, whereas PD098059 had no considerable effect (Figure 3C,D). These data suggest that activation of the Akt/Src/Cav1/catalase axis appears to be an essential prerequisite for the adaptation process leading to the development of radioresistance.

### 3.3. Statin Treatment Induces Radio- and Chemosensitization through Downregulation of Akt1/Cav1 Signaling and Increased Levels of Oxidative Stress

**Figure 4:**
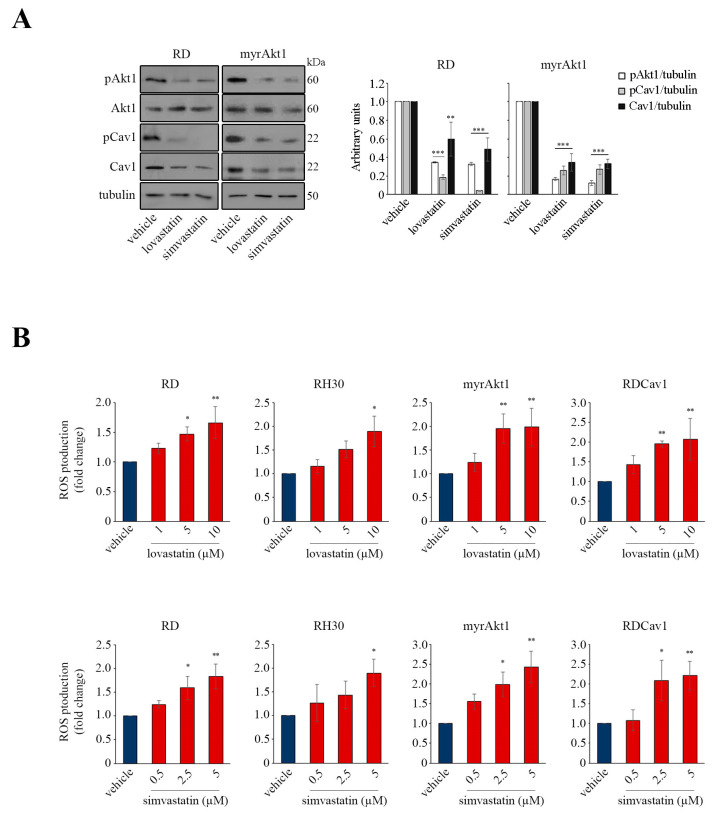
Statins downregulate Akt1 and Cav1 protein levels in myrAkt1 cells and increase ROS production in all RMS lines. (**A**) Cells were treated for 4 h with statins or vehicle 24 h after plating control and myrAkt1 cells (1.5 *×* 10^5^) in 60 mm dishes, then processed by IB analysis (*n* = 2). Densitometric quantifications are reported in the graphs after tubulin normalization. Data are mean ± SEM, ** *p*-value < 0.001; *** *p*-value < 0.0001; one-way ANOVA test vs. vehicle-treated cells. (**B**) Cells (3 × 10^3^) were plated in black 96-well plates in triplicates. ROS production was evaluated by administering the CM-H2DCFDA probe to all the indicated RMS lines after receiving statins or vehicle at the indicated doses for 24 h (*n* = 2). Data are mean ± SEM, * *p*-value < 0.05; ** *p*-value < 0.001; one-way ANOVA test vs. vehicle-treated cells.

**Figure 5:**
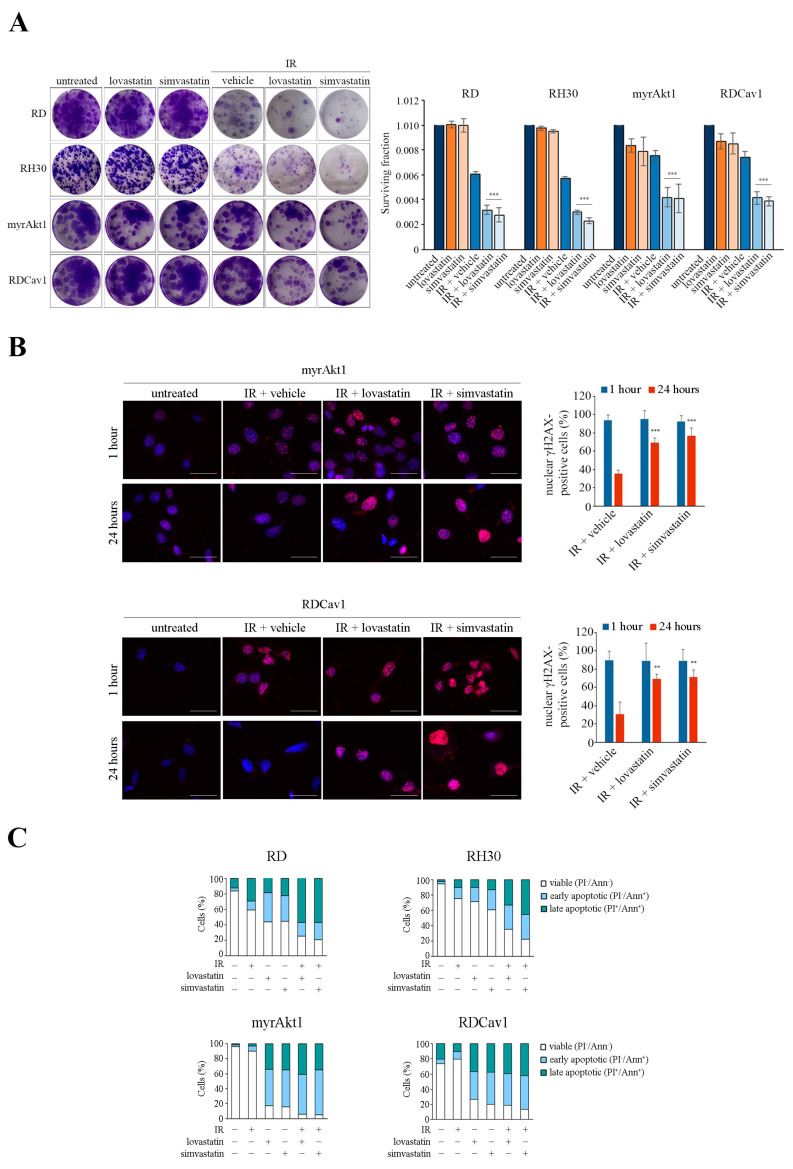
The combination of statins and IR promotes radiosensitization in RMS lines. (**A**) Subconfluent cells from RD, RH30, myrAkt1, and RDCav1 lines were treated for 2 h with statins or vehicle before IR treatment. Cell survival was evaluated by clonogenic assay (*n* = 2). Data are mean ± SEM, *** *p*-value < 0.0001; one-way ANOVA test vs. vehicle-treated irradiated cells. Representative images were taken after crystal violet incorporation. (**B**) IF analysis of nuclear γH2AX staining in myrAkt1 and RDCav1 cells pretreated for 2 h with statins or vehicle prior to IR and evaluated at the indicated time points using a fluorescent microscope at 63 X magnification. The scale bar corresponds to 50 µm. The quantification is relative to the average number of γH2AX-positive cells counted in ten different fields (*n* = 2). Data are mean ± SEM, ** *p*-value < 0.001; *** *p*-value < 0.0001; one-way ANOVA test vs. vehicle-treated irradiated cells for each time-point. (**C**) Cells (4 × 10^4^ for myrAkt1 and RDCav1, or 6 × 10^4^ for RD and RH30) were plated in duplicates into 6-well plates. FACS analysis was performed on all RMS lines after treatment with lovastatin or simvastatin and IR for 72 h (*n* = 2).

**Figure 6:**
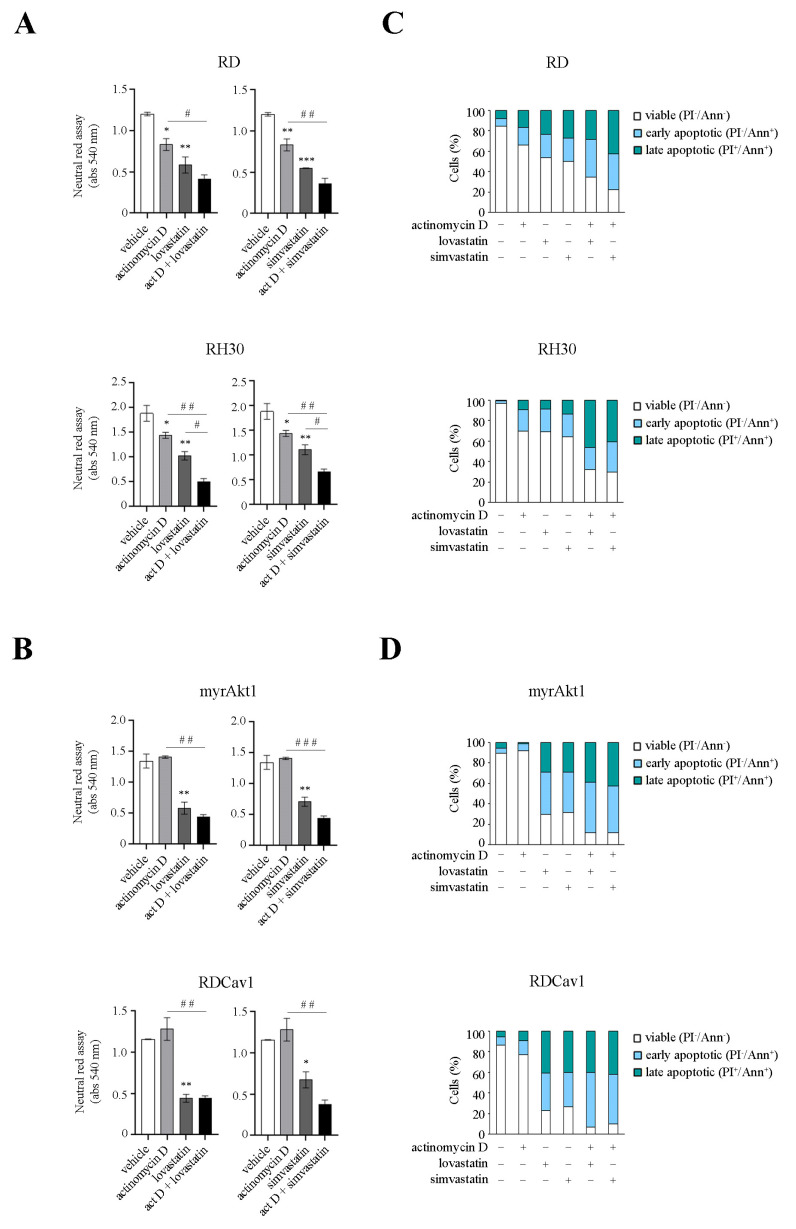
The combination of statins and actinomycin D promotes chemosensitization in RMS lines. (**A**,**B**) Cells (1.5 × 10^3^ for myrAkt1 and RDCav1, or 2 × 10^3^ for RD and RH30) were seeded in triplicates in 96-well plates 24 h prior to treatment with the indicated compounds. Cell survival was evaluated by neutral red assay after 48 h (*n* = 2). Data are mean ± SEM, * *p*-value < 0.05; ** *p*-value < 0.001; *** *p*-value < 0.0001; one-way ANOVA test vs. vehicle-treated cells. # *p*-value < 0.05; ## *p*-value < 0.001; ### *p*-value < 0.0001; one-way ANOVA test vs. single treatment. (**C**,**D**) Cells (4 × 10^4^ for myrAkt1 and RDCav1 or 6 × 10^4^ for RD and RH30) were plated in duplicates into 6-well plates. FACS analysis was performed on all RMS lines after treatment with lovastatin or simvastatin and actinomycin D for 72 h (*n* = 2).

Many studies have indicated that oxidative stress is involved in the various toxicities associated with statins [28]. In addition, we recently showed that activation of Akt1 increases susceptibility to statin treatment [9]. Furthermore, the localization and function of Cav1 are strictly dependent on the proper availability of cholesterol [15]. For these reasons, we decided to evaluate the impact of statin treatment on Akt1 signaling. In RD and myrAkt1 cells treated with lovastatin or simvastatin, both total and phosphorylated forms of Akt1 and Cav1 were significantly reduced as early as 4 h, as demonstrated by IB analysis (Figure 4A). Therefore, we decided to evaluate the ROS levels in statin-treated cells. As shown in Figure 4B, lovastatin or simvastatin dose-dependently increased intracellular ROS levels in RD and RH30 lines and in both radioresistant RMS lines as detected after 24 h. This evidence led us to hypothesize that statin treatment may result in attenuation of radioresistance mechanisms by decreasing the Akt1 pathway while simultaneously increasing ROS levels. To this end, the RMS lines were pretreated with lovastatin and simvastatin for 2 h before IR treatment. In the clonogenic assay, we observed that statins significantly reduced the cell viability of post-irradiated RD and RH30 lines as well as of radioresistant myrAkt1 and RDCav1 clones (Figure 5A). Statin-induced radiosensitization was confirmed by assessing γH2AX, a marker of DNA double strand breaks (DSBs). In radioresistant myrAkt1 and RDCav1 lines, IR treatment already promoted the appearance of nuclear staining indicative of DSBs after 1 h, which completely disappeared after 24 h, indicative of DNA repair (Figure 5B). However, treatment of cells with statins prior to IR resulted in prolonged γH2AX staining which was still detectable 24 h after IR, indicative of unrepaired DNA damage (Figure 5B). Using FACS analysis, we found that statins significantly increased cell apoptosis in all RMS lines (Figure 5C). Notably, statin treatment produced the strongest effect on the radioresistant lines, resulting in a percentage of cell apoptosis between 80 and 90% (Figure 5C). The combination of statins and IR significantly increased cell apoptosis in the RD and RH30 lines, while the additive effect in the radioresistant lines was less evident due to the strong effect already produced by statin treatment alone (Figure 5C). Because RMS therapy is based on the use of a multiagent cocktail including vincristine, actinomycin D, and cyclophosphamide (VAC), we tested the combination of statins and actinomycin D. Although actinomycin D is an inhibitor of RNA synthesis, it has been reported to act as an oxidative stress inducer as well [29]. Interestingly, actinomycin D treatment reduced cell survival and increased apoptosis in the RD and RH30 lines but not in the radioresistant lines, as evaluated by neutral red assay (Figure 6A,B) and FACS analysis (Figure 6C,D). The combination of statins and actinomycin D led to chemosensitization, impairing cell viability (Figure 6A,B) and increasing apoptosis (Figure 6C,D) in all cell lines. Overall, these data suggest that statin treatment may be critical for promoting radio- and chemosensitization in RMS cells.

Many studies have indicated that oxidative stress is involved in the various toxicities associated with statins [28]. In addition, we recently showed that activation of Akt1 increases susceptibility to statin treatment [9]. Furthermore, the localization and function of Cav1 are strictly dependent on the proper availability of cholesterol [15]. For these reasons, we decided to evaluate the impact of statin treatment on Akt1 signaling. In RD and myrAkt1 cells treated with lovastatin or simvastatin, both total and phosphorylated forms of Akt1 and Cav1 were significantly reduced as early as 4 h, as demonstrated by IB analysis (Figure 4A). Therefore, we decided to evaluate the ROS levels in statin-treated cells. As shown in Figure 4B, lovastatin or simvastatin dose-dependently increased intracellular ROS levels in RD and RH30 lines and in both radioresistant RMS lines as detected after 24 h. This evidence led us to hypothesize that statin treatment may result in attenuation of radioresistance mechanisms by decreasing the Akt1 pathway while simultaneously increasing ROS levels. To this end, the RMS lines were pretreated with lovastatin and simvastatin for 2 h before IR treatment. In the clonogenic assay, we observed that statins significantly reduced the cell viability of post-irradiated RD and RH30 lines as well as of radioresistant myrAkt1 and RDCav1 clones (Figure 5A). Statin-induced radiosensitization was confirmed by assessing γH2AX, a marker of DNA double strand breaks (DSBs). In radioresistant myrAkt1 and RDCav1 lines, IR treatment already promoted the appearance of nuclear staining indicative of DSBs after 1 h, which completely disappeared after 24 h, indicative of DNA repair (Figure 5B). However, treatment of cells with statins prior to IR resulted in prolonged γH2AX staining which was still detectable 24 h after IR, indicative of unrepaired DNA damage (Figure 5B). Using FACS analysis, we found that statins significantly increased cell apoptosis in all RMS lines (Figure 5C). Notably, statin treatment produced the strongest effect on the radioresistant lines, resulting in a percentage of cell apoptosis between 80 and 90% (Figure 5C). The combination of statins and IR significantly increased cell apoptosis in the RD and RH30 lines, while the additive effect in the radioresistant lines was less evident due to the strong effect already produced by statin treatment alone (Figure 5C). Because RMS therapy is based on the use of a multiagent cocktail including vincristine, actinomycin D, and cyclophosphamide (VAC), we tested the combination of statins and actinomycin D. Although actinomycin D is an inhibitor of RNA synthesis, it has been reported to act as an oxidative stress inducer as well [29]. Interestingly, actinomycin D treatment reduced cell survival and increased apoptosis in the RD and RH30 lines but not in the radioresistant lines, as evaluated by neutral red assay (Figure 6A,B) and FACS analysis (Figure 6C,D). The combination of statins and actinomycin D led to chemosensitization, impairing cell viability (Figure 6A,B) and increasing apoptosis (Figure 6C,D) in all cell lines. Overall, these data suggest that statin treatment may be critical for promoting radio- and chemosensitization in RMS cells.

Many studies have indicated that oxidative stress is involved in the various toxicities associated with statins [28]. In addition, we recently showed that activation of Akt1 increases susceptibility to statin treatment [9]. Furthermore, the localization and function of Cav1 are strictly dependent on the proper availability of cholesterol [15]. For these reasons, we decided to evaluate the impact of statin treatment on Akt1 signaling. In RD and myrAkt1 cells treated with lovastatin or simvastatin, both total and phosphorylated forms of Akt1 and Cav1 were significantly reduced as early as 4 h, as demonstrated by IB analysis (Figure 4A). Therefore, we decided to evaluate the ROS levels in statin-treated cells. As shown in Figure 4B, lovastatin or simvastatin dose-dependently increased intracellular ROS levels in RD and RH30 lines and in both radioresistant RMS lines as detected after 24 h. This evidence led us to hypothesize that statin treatment may result in attenuation of radioresistance mechanisms by decreasing the Akt1 pathway while simultaneously increasing ROS levels. To this end, the RMS lines were pretreated with lovastatin and simvastatin for 2 h before IR treatment. In the clonogenic assay, we observed that statins significantly reduced the cell viability of post-irradiated RD and RH30 lines as well as of radioresistant myrAkt1 and RDCav1 clones (Figure 5A). Statin-induced radiosensitization was confirmed by assessing γH2AX, a marker of DNA double strand breaks (DSBs). In radioresistant myrAkt1 and RDCav1 lines, IR treatment already promoted the appearance of nuclear staining indicative of DSBs after 1 h, which completely disappeared after 24 h, indicative of DNA repair (Figure 5B). However, treatment of cells with statins prior to IR resulted in prolonged γH2AX staining which was still detectable 24 h after IR, indicative of unrepaired DNA damage (Figure 5B). Using FACS analysis, we found that statins significantly increased cell apoptosis in all RMS lines (Figure 5C). Notably, statin treatment produced the strongest effect on the radioresistant lines, resulting in a percentage of cell apoptosis between 80 and 90% (Figure 5C). The combination of statins and IR significantly increased cell apoptosis in the RD and RH30 lines, while the additive effect in the radioresistant lines was less evident due to the strong effect already produced by statin treatment alone (Figure 5C). Because RMS therapy is based on the use of a multiagent cocktail including vincristine, actinomycin D, and cyclophosphamide (VAC), we tested the combination of statins and actinomycin D. Although actinomycin D is an inhibitor of RNA synthesis, it has been reported to act as an oxidative stress inducer as well [29]. Interestingly, actinomycin D treatment reduced cell survival and increased apoptosis in the RD and RH30 lines but not in the radioresistant lines, as evaluated by neutral red assay (Figure 6A,B) and FACS analysis (Figure 6C,D). The combination of statins and actinomycin D led to chemosensitization, impairing cell viability (Figure 6A,B) and increasing apoptosis (Figure 6C,D) in all cell lines. Overall, these data suggest that statin treatment may be critical for promoting radio- and chemosensitization in RMS cells.

Many studies have indicated that oxidative stress is involved in the various toxicities associated with statins [28]. In addition, we recently showed that activation of Akt1 increases susceptibility to statin treatment [9]. Furthermore, the localization and function of Cav1 are strictly dependent on the proper availability of cholesterol [15]. For these reasons, we decided to evaluate the impact of statin treatment on Akt1 signaling. In RD and myrAkt1 cells treated with lovastatin or simvastatin, both total and phosphorylated forms of Akt1 and Cav1 were significantly reduced as early as 4 h, as demonstrated by IB analysis (Figure 4A). Therefore, we decided to evaluate the ROS levels in statin-treated cells. As shown in Figure 4B, lovastatin or simvastatin dose-dependently increased intracellular ROS levels in RD and RH30 lines and in both radioresistant RMS lines as detected after 24 h. This evidence led us to hypothesize that statin treatment may result in attenuation of radioresistance mechanisms by decreasing the Akt1 pathway while simultaneously increasing ROS levels. To this end, the RMS lines were pretreated with lovastatin and simvastatin for 2 h before IR treatment. In the clonogenic assay, we observed that statins significantly reduced the cell viability of post-irradiated RD and RH30 lines as well as of radioresistant myrAkt1 and RDCav1 clones (Figure 5A). Statin-induced radiosensitization was confirmed by assessing γH2AX, a marker of DNA double strand breaks (DSBs). In radioresistant myrAkt1 and RDCav1 lines, IR treatment already promoted the appearance of nuclear staining indicative of DSBs after 1 h, which completely disappeared after 24 h, indicative of DNA repair (Figure 5B). However, treatment of cells with statins prior to IR resulted in prolonged γH2AX staining which was still detectable 24 h after IR, indicative of unrepaired DNA damage (Figure 5B). Using FACS analysis, we found that statins significantly increased cell apoptosis in all RMS lines (Figure 5C). Notably, statin treatment produced the strongest effect on the radioresistant lines, resulting in a percentage of cell apoptosis between 80 and 90% (Figure 5C). The combination of statins and IR significantly increased cell apoptosis in the RD and RH30 lines, while the additive effect in the radioresistant lines was less evident due to the strong effect already produced by statin treatment alone (Figure 5C). Because RMS therapy is based on the use of a multiagent cocktail including vincristine, actinomycin D, and cyclophosphamide (VAC), we tested the combination of statins and actinomycin D. Although actinomycin D is an inhibitor of RNA synthesis, it has been reported to act as an oxidative stress inducer as well [29]. Interestingly, actinomycin D treatment reduced cell survival and increased apoptosis in the RD and RH30 lines but not in the radioresistant lines, as evaluated by neutral red assay (Figure 6A,B) and FACS analysis (Figure 6C,D). The combination of statins and actinomycin D led to chemosensitization, impairing cell viability (Figure 6A,B) and increasing apoptosis (Figure 6C,D) in all cell lines. Overall, these data suggest that statin treatment may be critical for promoting radio- and chemosensitization in RMS cells.

## 4. Discussion

**Figure 7:**
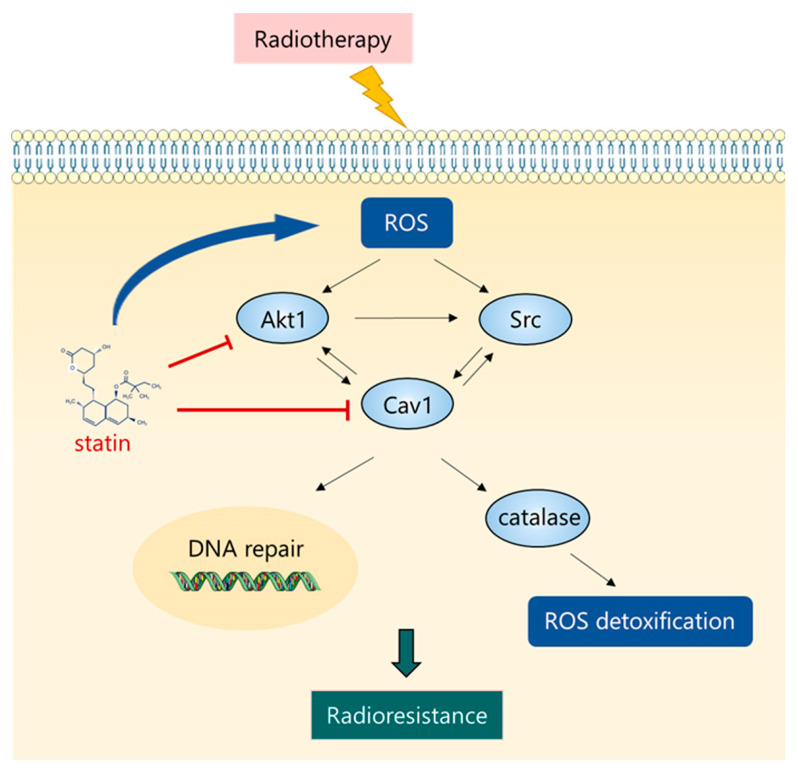
The Akt1/Src/Cav1 network increases ROS detoxification and DNA repair in RMS cells. Representative scheme of the protein network that facilitates ROS neutralization through increased catalase expression and DNA repair. Statin treatment induces high levels of ROS and radiosensitization by reducing Akt1 and Cav1 activation.

The development of new combination therapies for RMS is of great interest for circumventing resistance mechanisms and improving clinical efficacy [30]. In the present study, we show that inhibition and hyperactivation of the Akt/Src/Cav1 signaling pathway respectively increase and decrease the oxidative stress on RMS cells, suggesting that modulation of this pathway may play a crucial role in protection and sensitization to IR therapy. When intracellular ROS levels increase markedly, as occurs in response to radiotherapy, activation of this pathway appears to play a crucial role in facilitating ROS detoxification to promote cell survival. As illustrated in Figure 7, we propose a model in which the survival of irradiated cells is favored in a Darwinian manner through the activation of Akt1, which, by orchestrating the increased expression of Src and Cav1, fuels a positive regulatory mechanism that facilitates its own phosphorylation. This improves resistance to IR-induced oxidative stress and repair of DNA damage, as documented by increased catalase expression, lower levels of ROS, and reduction of DSBs. It should be noted that increased phosphorylation of Akt1, Src, and Cav1 was detected in the irradiated RH30 FPRMS line, suggesting that the antioxidant role of this signaling might be conserved in most radioresistant RMS, although this aspect requires further investigation.

ROS generation is one of the major mechanisms responsible for the therapeutic effect of IR and almost 50% of chemotherapeutic drugs. Like many other cancers, RMS is considered a tumor with high oxidative stress [31]. Activation of Akt and Src signaling after radiotherapy has been frequently detected in cancer [32], and Akt1 is considered to be a key determinant of intrinsic resistance in several cancer types [33]. Here, we show for the first time that Src and Cav1 are both downstream targets of Akt1 in RD cells. Based on this, we assume that Akt1 increases the phosphorylated form of Cav1 through a mechanism that is known to be Src-kinase-dependent [17,34]. It has been shown that Src-kinase activates and phosphorylates Cav1 under conditions of oxidative stress, suggesting that Src/Cav1 may act as sensors of oxidative stress [35]. In its phosphorylated form, Cav1 can move from the plasma membrane, where it binds a plethora of proteins, to other cell compartments, thereby producing altered internalization and trafficking of numerous molecules, including a variety of RTKs and G-protein-coupled receptors [35,36]. According to our data, the Akt1/Src/Cav1 signaling in RMS that activates in response to high oxidative stress seems to represent a key determinant of radioresistance by increasing ROS detoxification and repair of DNA damage, as previously anticipated in other works [23,37]. In this regard, the elevated catalase expression observed following activation of the Akt1/Src/Cav1 axis likely reflects an increased need for irradiated RMS cells to detoxify superoxide anions and the dismutation product H_2_O_2_ [27]. Interestingly, increased Cav1 expression in lung adenocarcinoma has been reported to attenuate H_2_O_2_ production favoring tumor progression [38]. Therefore, understanding the mechanisms through which the Akt1/Src/Cav1 pathway contributes to buffering oxidative stress and DNA repair remains one of the major objectives deserving of further investigation for the development of therapies aimed at overcoming resistance mechanisms in RMS.

The mechanisms by which oxidative stress in RMS can preferentially activate intracellular kinases have not been investigated thus far. It has been reported that high ROS levels can lead to activation of Src and Akt by reversible oxidation of conserved cysteine residues [39,40]. For example, ROS generated by IR therapy or by the activity of nicotinamide adenine dinucleotide phosphate hydrogen (NADPH) oxidases (NOX) and mitochondria can reversibly oxidate critical cysteine residues within Src, promoting its activation [41]. Similarly, prolonged phosphorylation of the PI3K/Akt pathway may result from IR-induced oxidative stress that mediates the inactivation of protein tyrosine phosphatases (PTPs) involved in dephosphorylation of RTK [42], such as erythroblastic oncogene B (ErbB)/human epidermal growth factor receptors (HER) [43]. Importantly, Akt can also be activated in response to IR-induced DNA damage by members of the phosphatidylinositol 3-kinase-related kinase (PIKK) family, including ataxia telangiectasia mutated (ATM), ataxia telangiectasia and Rad3-related protein (ATR), and DNA-PK, as a part of pro-survival program [44,45,46]. Therefore, it is of considerable importance to understand the mechanisms involved in the activation of antioxidant kinases, such as the Akt and Src kinases, in order to develop targeted therapies for RMS.

Previous works have shown that statins cause cytotoxicity in RMS cells by increasing autophagy and apoptosis [47,48,49]. We have confirmed these data here, further demonstrating that statin treatment generates high oxidative stress in RMS cells, leading to cell apoptosis. In this regard, the excessive or long-term use of statins is known to cause in vitro and in vivo cytotoxicity that seems to be mainly related to oxidative stress [28], as observed in skeletal muscle, where rhabdomyolysis may occur [50]. In RD and myrAkt1 cells, the Akt1 and Cav1 proteins were robustly downregulated after statin treatment. We do not know the mechanism by which statins induce Akt1/Cav1 downregulation; nevertheless, statin-mediated attenuation of the Akt and mTOR signaling pathways has already been correlated with reduction of tumor burden in different cancers [51]. We assume that, after statin treatment, the failure to activate the Akt1/Cav1 pathway determines the inability to buffer oxidative stress, facilitating ROS accumulation and apoptotic events. Interestingly, the radioresistant myrAkt1 and RDCav1 lines were more protected from the cytotoxic effects of actinomycin D, a classic antineoplastic agent used in the treatment of RMS that primarily acts as an inhibitor of RNA synthesis but which also induces oxidative stress [29]. Statin treatment in combination with IR or actinomycin D was effective to enhance radio- and chemosensitization of RMS lines. To explain the effects of statins, we must remember that statins, as inhibitors of 3-hydroxy-3-methylglutaryl-coenzyme A reductase, reduce the levels of total cholesterol as well as of several isoprenoid intermediates, including farnesyl pyrophosphate (FPP) and geranylgeranyl pyrophosphate (GPP), which are necessary for prenylation of many proteins, including small GTP-binding proteins (GTPases) such as the Ras superfamily and Rho family of proteins [52]. As a result of reduced post-translational protein prenylation, statins may interfere with a plethora of biochemical mechanisms that are essential to cell survival, signaling, and proliferation. In addition, several studies have indicated that cholesterol reduction at the plasma membrane affects the spatial localization and activation of the PI3K/Akt pathway and Cav1, an event that may exert anti-cancer effects, as reviewed in [53]. Therefore, targeting the cholesterol metabolism using in vivo RMS models and tumor xenografts will contribute to a better understanding of the statin efficacy.

The present study has a number of limitations. First, the data provided for the antioxidant role of the Akt1/Src/Cav1 signaling should be extended to additional RMS lines. The second important issue concerns the analysis of the effects induced by antioxidant agents. These aspects certainly deserve future investigation.

## 5. Conclusions

The results presented here shed light on the existence of adaptive Akt1/Src/Cav1 signaling which confers radioresistance in RMS. Our data indicate that the Akt pathway acts synergistically with Cav1 and Src to promote ROS detoxification and DNA repair. Importantly, this pathway appears to be severely affected by treatment with statins, which could be used to restore radiosensitization. This molecularly linked signature may represent a prognostic determinant for identifying subsets of RMS patients with higher probability of relapse after radiotherapy.

## Data Availability

The data presented in this study are openly available in the NCBI Gene Expression Omnibus database (accession number GSE108022) at 10.1158/2159-8290.CD-13-0639, reference number [4].

## Supplementary Materials

The following are available online at: https://www.mdpi.com/2072-6694/16/5/853/s1, File S1: Original Western Blot images.
