# Supplementary figures and images for: Statin-Sensitive Akt1/Src/Caveolin-1 Signaling Enhances Oxidative Stress Resistance in Rhabdomyosarcoma
Source: Cancers (Basel). 2024 Feb 20;16(5):853. doi: 10.3390/cancers16050853 (PMC11154391; doi:10.3390/cancers16050853)

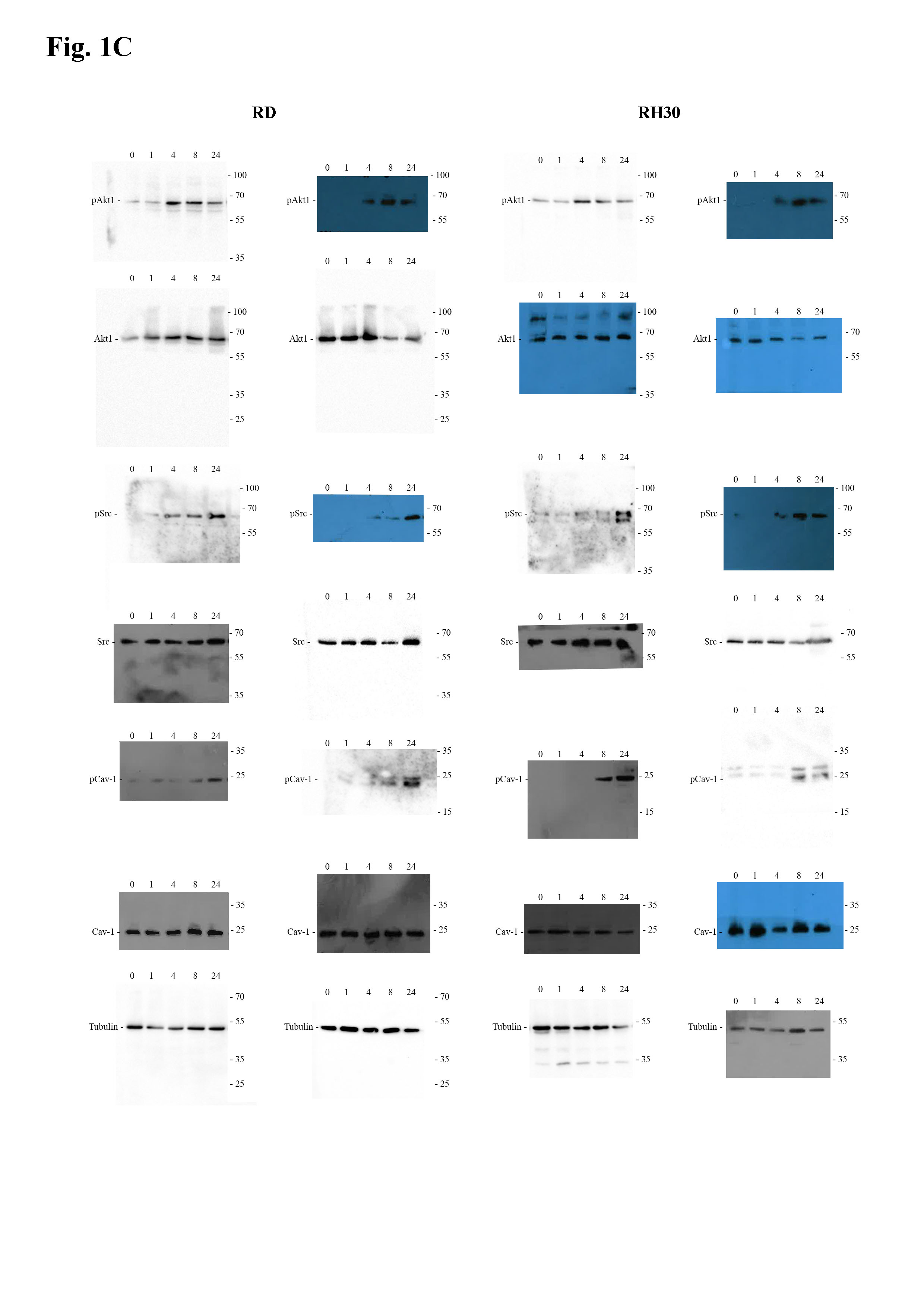

Supplement: Supplementary file 1 [file cancers-16-00853-s001.zip › Original blot Fig 1C NEW.jpg]

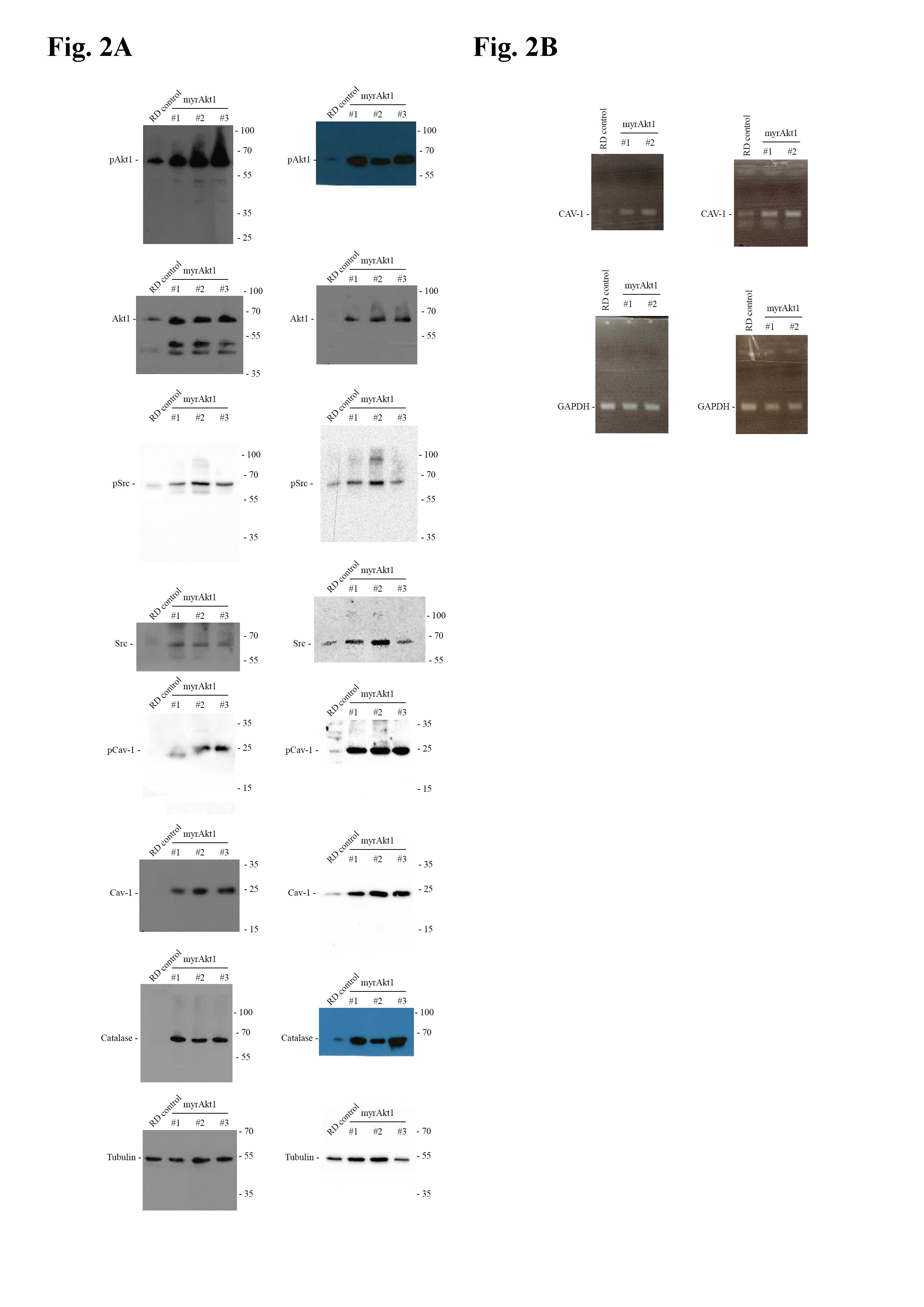

Supplement: Supplementary file 1 [file cancers-16-00853-s001.zip › Original blot Fig 2A, B NEW.jpg]

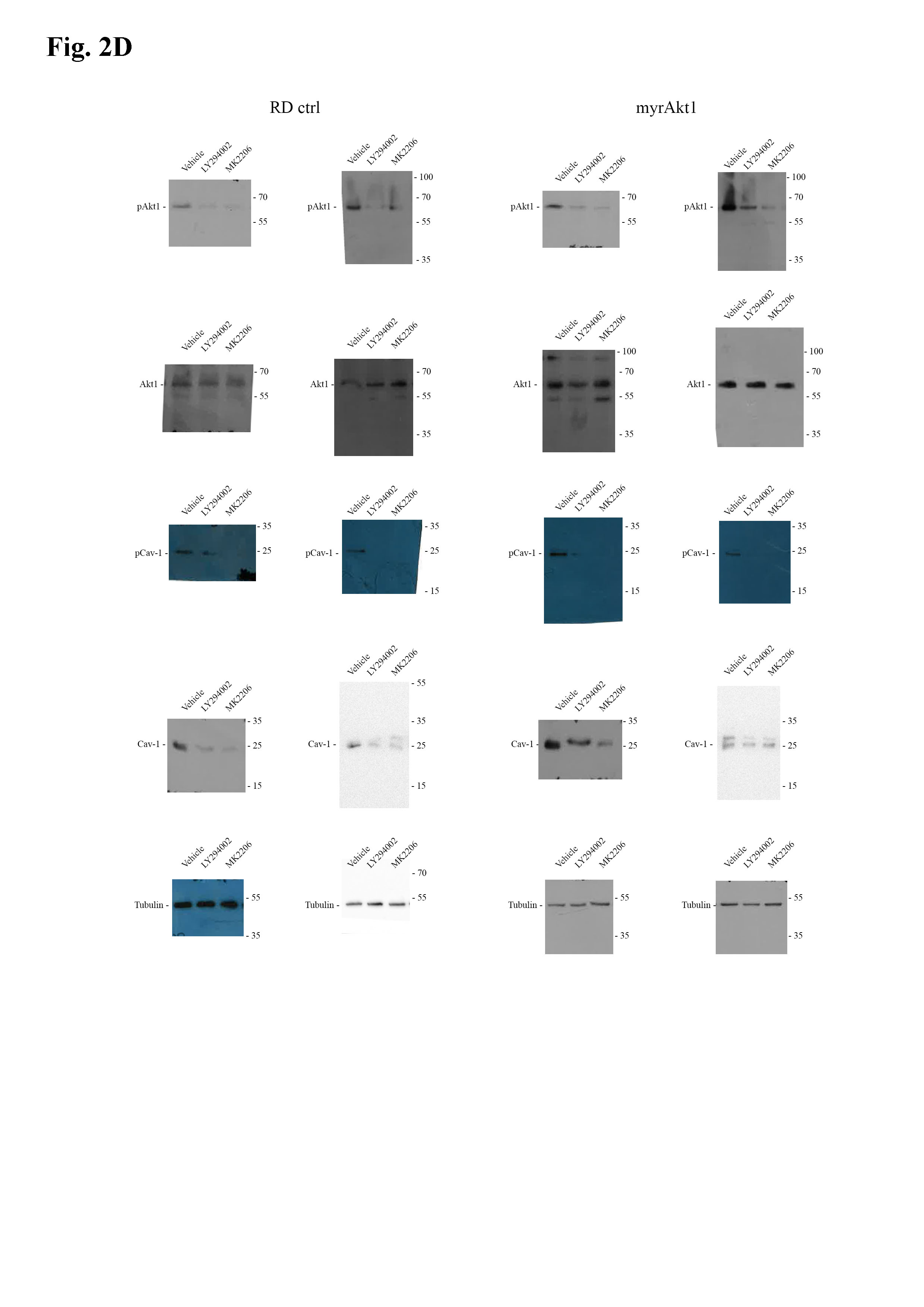

Supplement: Supplementary file 1 [file cancers-16-00853-s001.zip › Original blot Fig. 2D NEW.jpg]

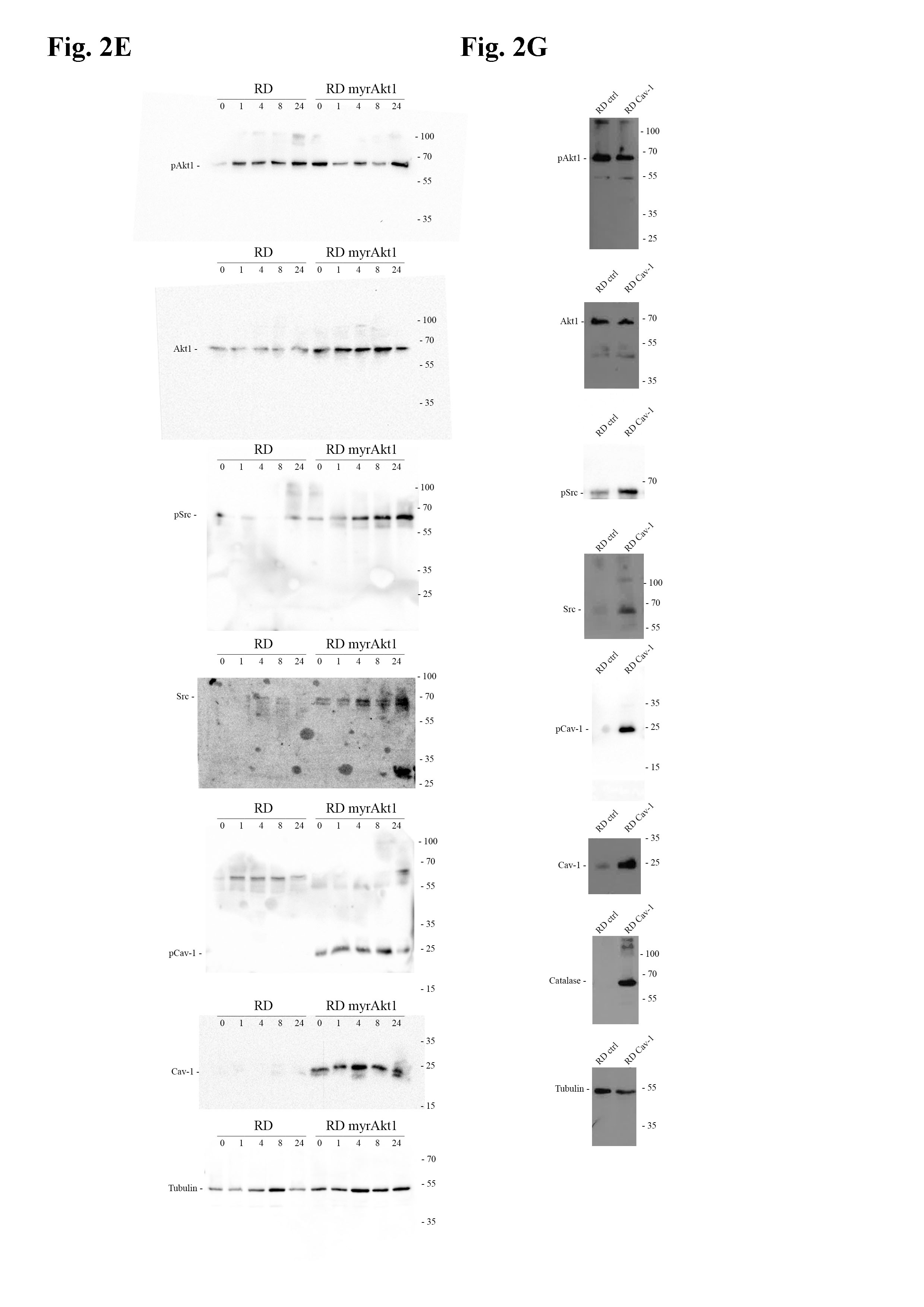

Supplement: Supplementary file 1 [file cancers-16-00853-s001.zip › Original blot Fig. 2E, G NEW.jpg]

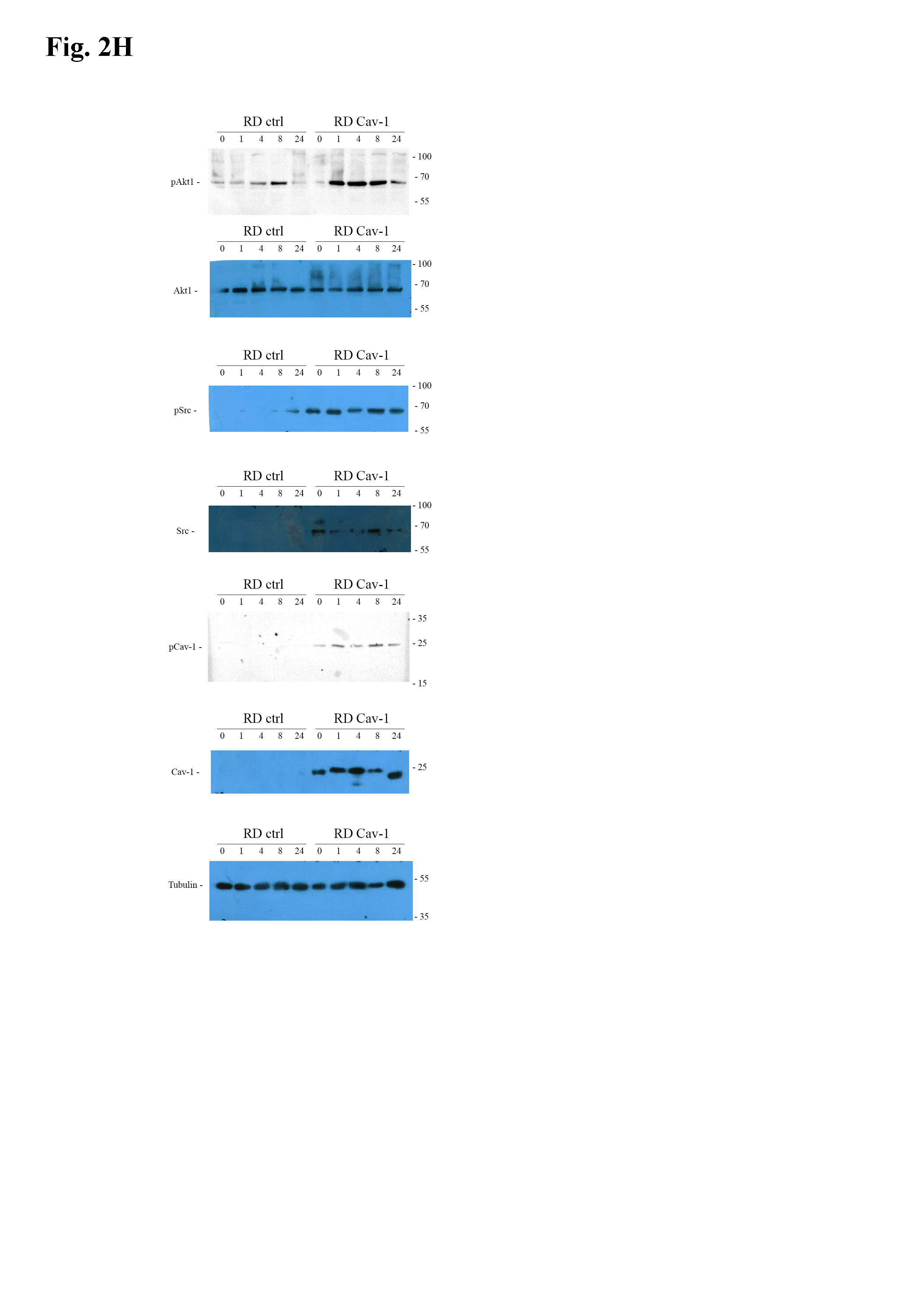

Supplement: Supplementary file 1 [file cancers-16-00853-s001.zip › Original blot Fig. 2H NEW.jpg]

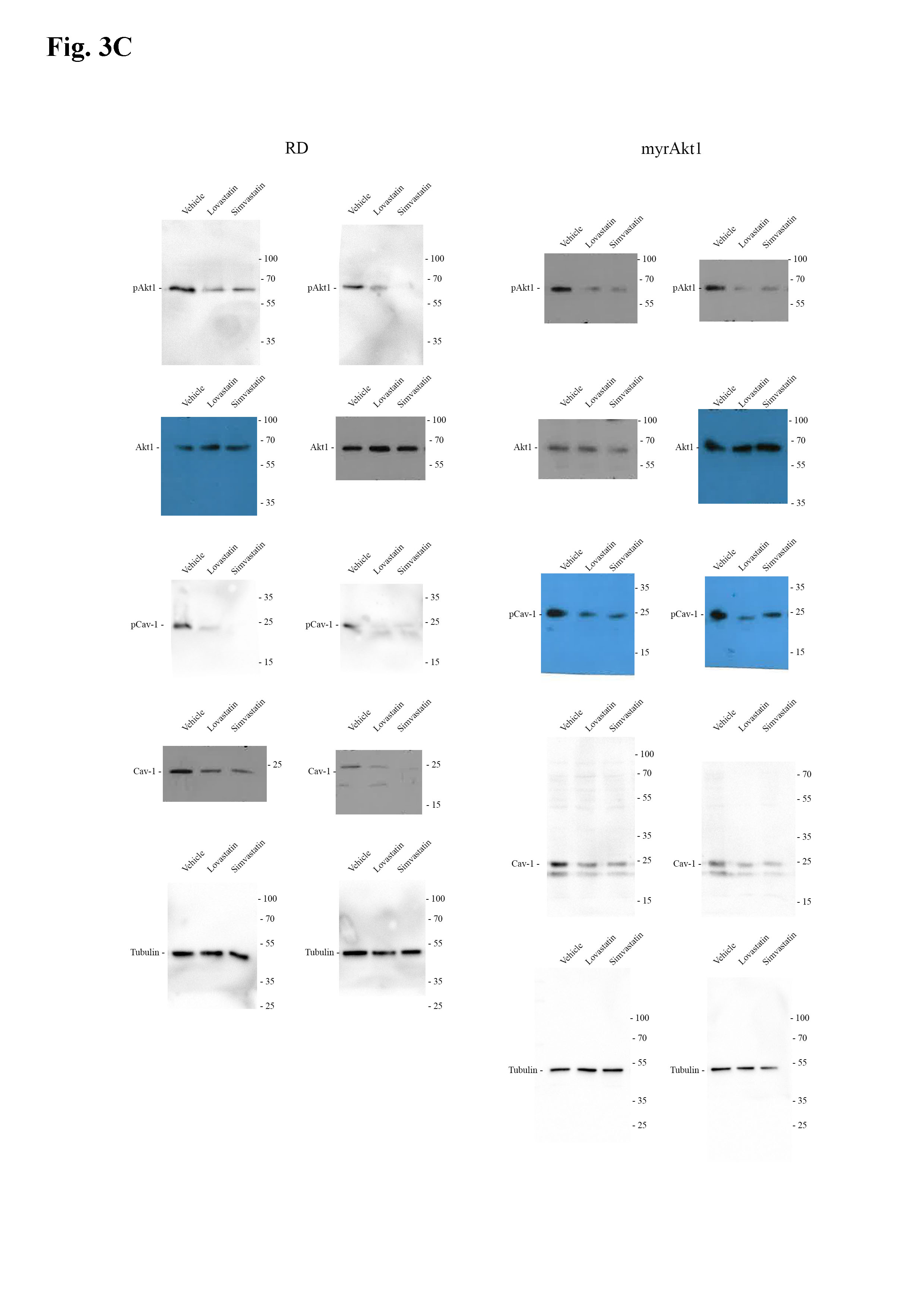

Supplement: Supplementary file 1 [file cancers-16-00853-s001.zip › Original blot Fig. 3C NEW.jpg]
